# Cross-Linked Gelatine by Modified Dextran as a Potential Bioink Prepared by a Simple and Non-Toxic Process

**DOI:** 10.3390/polym14030391

**Published:** 2022-01-19

**Authors:** Lenka Musilová, Eva Achbergerová, Lenka Vítková, Roman Kolařík, Martina Martínková, Antonín Minařík, Aleš Mráček, Petr Humpolíček, Jiří Pecha

**Affiliations:** 1 Department of Physics and Materials Engineering, Faculty of Technology, Tomas Bata University in Zlin, Vavreckova 275, 760 01 Zlín, Czech Republic; lmusilova@utb.cz (L.M.); vitkova@utb.cz (L.V.); minarik@utb.cz (A.M.); 2 Centre of Polymer Systems, Tomas Bata University in Zlín, tř. Tomáše Bati 5678, 760 01 Zlín, Czech Republic; rkolarik@utb.cz (R.K.); martinkova@utb.cz (M.M.); humpolicek@utb.cz (P.H.); 3 CEBIA-Tech, Faculty of Applied Informatics, Tomas Bata University in Zlín, Nad Stráněmi 4511, 760 05 Zlín, Czech Republic; achbergerova@utb.cz (E.A.); pecha@utb.cz (J.P.)

**Keywords:** gelatine-dextran, hydrogel, 3D printing, microextrusion, rheology, cell distribution

## Abstract

Essential features of well-designed materials intended for 3D bioprinting via microextrusion are the appropriate rheological behavior and cell-friendly environment. Despite the rapid development, few materials are utilizable as bioinks. The aim of our work was to design a novel cytocompatible material facilitating extrusion-based 3D printing while maintaining a relatively simple and straightforward preparation process without the need for harsh chemicals or radiation. Specifically, hydrogels were prepared from gelatines coming from three sources—bovine, rabbit, and chicken—cross-linked by dextran polyaldehyde. The influence of dextran concentration on the properties of hydrogels was studied. Rheological measurements not only confirmed the strong shear-thinning behavior of prepared inks but were also used for capturing cross-linking reaction kinetics and demonstrated quick achievement of gelation point (in most cases < 3 min). Their viscoelastic properties allowed satisfactory extrusion, forming a self-supported multi-layered uniformly porous structure. All gelatin-based hydrogels were non-cytototoxic. Homogeneous cells distribution within the printed scaffold was confirmed by fluorescence confocal microscopy. In addition, no disruption of cells structure was observed. The results demonstrate the great potential of the presented hydrogels for applications related to 3D bioprinting.

## 1. Introduction

Nowadays, 3D bioprinting has become one of the lead technologies in tissue engineering. Compared to the traditional preparation of cell-seeded scaffolds, the 3D bioprinting via microextrusion process enables the incorporation of selected cells within the printed material prior to or directly during the printing process. The major advantage of this technology is that cells, biomaterials, and biomolecules can be spatially defined. Therefore, more homogeneous cell distribution through the material could be achieved using this technique [1]. In addition, 3D bioprinting is more straightforward, less prone to human error, and gives an opportunity to precisely fabricate complex structures [2,3]. The technique relies on well-designed materials, so-called bioinks, which are essential for 3D-bioprinted scaffolds in tissue engineering [4]. Despite the rapid development, the discipline still has a shortage of materials utilizable as bioinks [5,6,7].

Materials for microextrusion in biological applications need to fulfill several criteria, concerning both cellular response to the ink and mechanical response to printing-induced stress. Good cytocompatibility and a suitable micro- and nanostructure serve to facilitate cell proliferation and growth [8,9,10]. Regarding the physical behavior during printing, their rheological properties present the main contribution. They have to be tailored in a way that allows uninterrupted flow of the material in the nozzle and provides stability to the printed structure at the same time. In addition, the bioink should help minimize the shear stress during printing in order to avoid the risk of cell destruction [11,12]. In addition to suitable rheology, sufficient layer adhesion is needed to ensure stable structures [13,14]. From the described point of view, hydrogels hold a great promise as potential bioinks [15]. Those formed from biopolymers such as hyaluronan (HA), collagen, or gelatine (Gel) are especially useful due to their ability to mimic the cellular environment [16].

Hydrogels generally consist of a cross-linked polymer network. The cross-linking can be facilitated either by non-covalent interactions or covalent (chemical) interactions. A typical feature of the former is the reversibility of bonds under specific conditions, which offers an opportunity in terms of rheology tuning [17,18]. The downside to this characteristic is the sensitivity to changes of thermodynamic conditions. It can also result in poor mechanical properties of the hydrogels [13,19,20]. In contrast, covalent bonds are less dynamic, but they provide the material with long-term stability in various environments. Furthermore, hydrogels cross-linked by this type of bond show superior durability under mechanical stress compared to non-covalent ones [17,21,22], which is desired for printed products [20].

To date, several chemically cross-linked hydrogels based on biopolymers for 3D bioprinting have been reported. Photocross-linking using UV irradiation was performed for modified natural polymers, such as HA [23] or in combination with modified polypeptides [16,24]. Although UV-initiated polymerization is popular due to its effectivity and predictability, this approach is also associated with the potential risk of inducing chromosomal and genetic instabilities in cells and subsequent cell mortality. The weaknesses of UV light were omitted when HA and Gel, both modified by phenolic hydroxyl moieties, were photocross-linked by irradiation from a visible spectrum. However, a disadvantage of this hydrogel preparation was the employment of a ruthenium/ammonium persulfate system [25]. Regarding other methods of bioink preparation, Gel–norbornene hydrogels were synthesized by two-photon polymerization [26], or modified HA, Gel, and acrylate cross-linked via radical polymerization [27] have been reported. Nevertheless, both mentioned methods required the complex chemical modification of used biopolymers before hydrogels preparation.

In a different approach, a dual cross-linking mechanism employing enzymatic reaction and photocross-linking [28,29] or photo- with chemical cross-linking [30] was utilised for bioink preparation [28,29,30]. Although the dual cross-linking strategies were developed to improve the mechanical and degradation properties of bioink while maintaining their printability and cell viability, this approach involves multi-step bioink preparation processes, especially when it is compared to much more straightforward simple methods utilizing UV irradiation [16,23,24]. On the other hand, the combined methods may allow avoiding the harmful effect of high-energy light by shifting the UV irradiation prior to embedding the cells in the material [29].

The aim of our work was to design a novel bipolymer-based hydrogel using chemical cross-linking hydrogels that allows microextrusion printing while maintaining a relatively simple and straightforward preparation process. Specifically, three types of Gel were examined: bovine (Gel-B)—a source of Gel often used in biomedical application—and two promising alternative sources—rabbit (Gel-R) and chicken (Gel-C) gelatines. The advantages of Gel-R and Gel-C are that they do not suffer from concerns about bovine spongiform encephalopathy or religious limitations [31,32]. All three gelatines were combined with dextran polyaldehyde (Dex-Ox) providing firm hydrogels. To the best of our knowledge, this presents a unique approach to using the described hydrogels (Gel-Dex-Ox) as a convenient material for 3D printing bearing the potential to be combined with living cells in a direct and simple procedure, thus creating a bioink.

Prepared printing materials were thoroughly investigated, and their performance in microextrusion-based 3D printing was evaluated. The study comprises hydrogels reaction kinetics, a detailed characterization of hydrogel rheological and swelling behavior, porosity, and printability, which are discussed with respect to Gel origin and the amount of cross-linking agent used throughout the study.

Die swell, a parameter closely connected to printing precision in microextrusion [14,33], is, to our best knowledge, underrepresented in case of biopolymer-based hydrogel 3D printing. Die swell is a result of normal stress induced by the sudden change in diameter of the flow channel [34]. Although the mechanical stress-induced cell mortality is primarily connected to tangential forces [35], evidence of normal stress affecting cell viability during printing have been found as well [36]. Consequently, the performed analysis also includes the issue of die swell.

Regarding the hydrogels performance in biomedical application, cytotoxicity assay was performed on the materials. Finally, fluorescently labeled mouse fibroblasts were added to the gels and printed so that the cell distribution could be evaluated. The prepared hydrogels proved to be shear thinning and suitable for 3D printing applications as well as showing good cytocompatibility and negligible deformation of cells during printing.

## 2. Materials and Methods

### 2.1. Chemicals

Gel-B (dry content 91.3%, M${}_{w}$ = 209,600 g·mol${}^{-1}$) and Gel-R (dry content 86.5%, M${}_{w}$ = 157,800 g·mol${}^{-1}$) were obtained from Tanex Vladislav, a.s. Gel-C (dry content 92.7%, M${}_{w}$ = 190,900 g·mol${}^{-1}$) was prepared according to a patented biotechnological process [37], which is described in detail in Mokrejš et al., 2019 [38] and Gál et al., 2020 [39]. Dextran (Dex) M${}_{w}$ = 40,400 g·mol${}^{-1}$, sodium periodate, and phosphate-buffered saline sterile solution (PBS), pH 7.4, were obtained from Sigma Aldrich. Demineralized (DEMI) water was prepared using Milipore Q System. Ammonia solution, 30 vol% was purchased from Penta and diluted to 25 vol%. Na${}_{2}$HPO${}_{4}$.12H${}_{2}$O and NaH${}_{2}$PO${}_{4}$.2H${}_{2}$O, used for the preparation of PBS pH 7, were obtained form Lach-Ner.

### 2.2. Dextran Oxidation

The oxidation of Dex was performed according to the previously described method [40]. Briefly, to the 13 wt % water solution of Dex and a 0.4 molar fold of NaIO${}_{4}$ pre-dissolved in 5 mL of DEMI water was added. The reaction was stirred for 4 h at room temperature. Subsequently, the reaction mixture was diluted with DEMI water and put into a dialysis tube (membrane cut-off 12,000 g·mol${}^{-1}$). The crude product was purified via dialysis against DEMI water for 3 days. Then, the solution was casted in a glass mold and frozen first at −18 ${}^{\circ }$C for 24 h followed by freeze drying in a freeze-dryer (ALPHA1-2 LD plus, M. Christ, Osterode am Harz, Germany). The pure product was obtained in yield 90%, and its M${}_{w}$ was 7700 g·mol${}^{-1}$. The number of aldehyde groups per 100 glucose subunits was determined using hydroxylamine hydrochloride method [41]. Automatic titrator T50 (Metler Tolledo, Greifensee, Switzerland) was used for the measurements.

### 2.3. Polymers Characterisation

Proton Nuclear Magnetic Resonance (${}^{1}$H NMR) spectra were recorded on a machine JEOL ECZ 400 (JEOL Ltd., Tokyo, Japan) operating at ${}^{1}$H frequency of 399.78 MHz at 60 ${}^{\circ }$C. The samples were dissolved in D${}_{2}$O at concentration of 10 mg·mL${}^{-1}$ for the analysis. The water signal was used as reference and was set at 4.75 ppm.

The average molecular weight and distribution curve of the initial biopolymers were determined by means of the size exclusion chromatography (SEC) method performed on a high-performance liquid chromatograph (HPLC) system Shimadzu Prominence equipped with UV-Vis and RI detectors (Shimadzu Prominence, LC-20 series, Shimadzu corporation, Kyoto, Japan). The conditions for analysis of polysaccharides were following: 0.1M PBS solution of pH equal to 7.4, flow 0.8 mL·min${}^{-1}$, oven temperature 30 ${}^{\circ }$C, columns PL aquagel-OH 60 8 μm, 300 × 7.5 mm and PL aquagel-OH 40 8 μm, 300 × 7.5 mm were connected in series. Pullulan standards were used for molecular weight calibration, analysis was based on RI data. Conditions for analysis of proteins were as follows: 0.15 M PBS solution of pH equal to 7.0, flow 0.35 mL·min${}^{-1}$, oven temperature 30 ${}^{\circ }$C, column Agilent Bio SEC-5, 5 μm, 150 Å, 300 × 4.6 mm. Protein standards were used for molecular weight calibration; analysis was based on UV data gained at 210 nm.

### 2.4. Hydrogels Preparation and Characterization

Hydrogels were prepared in the following manner: 2 wt % solution of Dex-Ox in PBS (0.1 M, pH 7.4) was mixed with 15 wt % solution of Gel dissolved in PBS (0.1 M, pH 7.4). Three volume ratios of the solutions were examined—Gel:Dex-Ox 1:1, 2:1, and 3:1. After that, 25 vol % ammonia solution was added in concentration 50 μL per 1 mL of Gel solution, and all the reactants were mixed.

Rheological measurements of the prepared fresh mixture of biopolymer solutions (2 mL) were performed on a rotational rheometer Anton-Paar MCR 502 (Graz, Austria) at 30 ${}^{\circ }$C under normal pressure in an air atmosphere. In case of the reaction kinetics measurement, time sweep experiments were performed using a 50 mm parallel-plate measuring system oscillating at constant 10% deformation with a constant angular frequency of 10 rad·s${}^{-1}$. Fundamental rheological data, i.e., complex viscosity η, storage (G${}^{\prime }$), and loss (G${}^{\prime \prime }$) moduli, were followed in a 40 min time sweep. It should be noted that the sample preparation caused a 1 min delay between the reaction start and first data obtained.

On the other hand, the rheology of fully cross-linked hydrogels was performed using a 25 mm parallel-plate measuring system oscillating at constant 10% deformation with angular frequency sweep increasing from 0.1 to 10 rad·s${}^{-1}$ at 35 °C. The frequency sweep measurement in a descending direction was carried out as well, without any change in rheological behavior. It is important to note that before such measurement was started, the hydrogel samples were prepared 12 h before in the form of circular plates with a diameter of 30 mm and a thickness of 2 mm.

As a 3D Printing instrument, Cellink BioX (Gothemburg, Sweden) was used with the following specifications: a polypropylene conical nozzle—0.41 mm diameter, 3 mL polypropylene syringe, microextrusion syringe pump printhead, and microscope glass slide printbed. The printhead speed was 2 mm·s${}^{-1}$, and the extrusion rate was 1.5 μL·s${}^{-1}$. During printing, both the printhead and printbed were kept at room temperature. Optical analysis of the printing performance was carried out using a Dino-Lite AM4815ZT optical microscope and evaluated with the aid of ImageJ software. The shape fidelity was characterized using the method described by Ouyang et al. [42], i.e., determining the printability (Pr) as the similitude of a gap between printed strands to a square in the top layer of a multi-layered 10x10 mm rectilinear patterned grid. The distance between strand centers in a single layer had to be adjusted to 3.3 mm due to the strong die swell of the material. The layer height was set to 0.6 mm in order to account for the die swell as well as to ensure good adhesion between layers. To calculate Pr, the following formula was used: Pr = L${}^{2}$/16A, where L denotes the perimeter (mm) and A the area of a gap (mm${}^{2}$). Moreover, an uninterrupted flow of material was recorded, and the die swell was measured at the perceived distance between the printbed and nozzle, i.e., 0.5 mm. Additionally, a model specifically designed for the materials examined in the current study was developed in the following way: The overall dimensions were 10 × 10 × 5 mm, the layer height was 1 mm, the material extrusion was continuous, and the speed of the printhead was monotonous throughout the printing.

The shape and porosity of printed structures before and after freeze drying was analyzed using X-ray computed micro-tomography (CT) with the help of SkyScan (Model 1174, Bruker, Billerica, MA, USA). The printed structures were obtained using the material-specific model described earlier. The device was equipped with the X-ray source, (voltage of 20–50 kV, maximum power of 40 W) and the X-ray detector. The CCD 1.3 Mpix was coupled to the scintillator by a lens with 1:6 zoom range. The projection images were recorded at angular increments of 0.5${}^{\circ }$ or 1${}^{\circ }$ using tube voltage and tube current of 35 kV and 585 μA, respectively. The exposure time was set to 15 s without using any filter. The 3D reconstructions, surface, and volume analysis were performed via built-in CT image analysis software (version 1.16.4.1, Bruker, USA). The results, in terms of images with different X-ray adsorption, 2D cross-sections, and 3D models were exported from DataViewer and CTvox software. Prior to CT characterization, the printed hydrogels were placed in a closed sample holder with increased humidity so that the analyzed scaffold does not dry out.

The inner porosity of the material was assessed via scanning electron microscopy (SEM) imaging of freeze-dried samples in vertical sections using a Phenom Pro instrument at an accelerating voltage of 10 kV. The samples were sputtered with a gold/palladium layer prior to imaging. The pore size and total pore area were statistically evaluated with the aid of ImageJ software.

The swelling behavior of hydrogels was determined gravimetrically as follows: weighed lyophilized samples were immersed in PBS (0.1 M pH 7.4) to gradually reach swelling equilibrium. The equilibrium buffer uptake, S(e)(%), of hydrogels was determined by taking the swollen samples from buffer solutions at selected time intervals of 1, 2, 6, 15, 30, 60, 120, 240, 360, and 1440 min, wiping with tissue paper and weighing. The presented results are expressed as an average values of 4 measurements. The samples were conditioned to 37 ${}^{\circ }$C throughout the measurement in order to meet the requirements of testing for biological use.

### 2.5. Cytotoxicity

Cytotoxicity was tested using a mouse embryonic fibroblast cell line (ATCC CRL-1658 NIH/3T3). Testing was performed according to ISO 10-993 standard concretely by testing of extracts from freeze-dried hydrogel samples. Extracts were prepared according to ISO standard 10993-12 with modifications; the extraction ratio was 0.02 g per 1 mL of culture medium (which is a lower amount than according to the ISO, which is due to the swelling properties of lyophilized samples). The ATCC-formulated Dulbecco’s Modified Eagle’s Medium (PAA Laboratories, Inc., Etobicoke, ON, Canada) containing 10% of calf serum (BioSera, Nuaille, France) and 100 U mL${}^{-1}$ penicillin/streptomycin (GE 209 Healthcare HyClone, Hyclone Ltd., Cramlington, UK) was used as the culture medium. Tested samples were extracted in culture medium for 24 h at 37 ${}^{\circ }$C under stirring. Subsequently, the extracts were filtered using a syringe filter with a pore size of 0.22 μm. Then, the parent extracts (100%) were diluted in culture medium to obtain a series of dilutions with concentrations of 75, 50, 25, 10, and 5%. Cells were proceeded in concentration of 10${}^{5}$ per 1 mL and cultivated for 24 h at 37 ${}^{\circ }$C in 5% CO${}_{2}$ in humidified air. Then, the medium was removed after the pre-cultivation and replaced by individual extracts. Cell viability was evaluated after 24 h of exposure using ATP assay (ATP Determination Kit A22066, ThermoFisher Scientific, Waltham, MA, USA). The results are presented as the relative cell viability compared to the reference (cells cultivated without extracts), where the reference corresponding to 1 means 100% cell viability. The presented data are from three experiments, each performed in triplicate.

### 2.6. Cell Distribution within 3D-Printed Structure

Before the test, the cells were fixed and counterstained. The 4% formaldehyde (Penta chemicals, Prague, Czech Republic) was used to fix the cells within the suspension. After 15 min of exposure, the cell suspension was centrifugated (1.5 RPM for 2 min) and supernatant was aspirated. Then, the cells were washed with PBS, and after centrifugation (1.5 RPM for 2 min), 0.5% Triton x-100 (Merck Group, Darmstadt, Germany) was added for 5 min followed by centrifugation and three washes with PBS. Then, the cells nuclei were counterstained by Hoechst 3325 ($\lambda_{ex}$ = 355 nm, $\lambda_{em}$ = 465 nm) and the cytoskeleton was counterstained by ActinRed 555 ($\lambda_{ex}$ = 540 nm and $\lambda_{em}$ = 665 nm) according to the protocol of the producer (both Sigma Aldrich). The stained fibroblasts were mixed with hydrogel in concentration of 5·10${}^{5}$ cells per 1 mL of hydrogel. These mixtures were printed (using the same procedure as describe before) and observed by the means of confocal microscopy using an Olympus FLUOVIEW FV3000 (Olympus corporation, Laser Scanning Confocal Microscope (LSCM) in order to determine the homogeneity of cells distribution. The Plan-Apochromat objective with magnification 10× and numerical aperture NA = 0.8 or 4× and NA = 0.4, respectively, were used for analysis. The figures were obtained as three-dimensional reconstruction from confocal images in the z-axis (4× magnification—10 images with 10 μm steps, 10× magnification—10 images with 5 μm steps).

## 3. Results and Discussion

### 3.1. Polysaccharide Oxidation and Hydrogel Formation

In order to develop printable hydrogels as potential bioinks based on a chemically cross-linked polymeric matrix, modified Dex and Gels were utilized. Bovine, rabbit, and chicken gelatines, hydrolyzed forms of collagens, were chosen in order to achieve close resemblance of the scaffold to extracellular matrix [43], thus maximizing the potential to produce material which may ensure sufficient viability, adhesion, and proliferation of fibroblasts [44]. Note that the source of gelatine and method of its preparation affect the ultimate mechanical and functional properties of final hydrogels [45], and consequently, the present study shall facilitate comparison of these Gel sources as a matrix of printable hydrogel.

**Figure 1:**
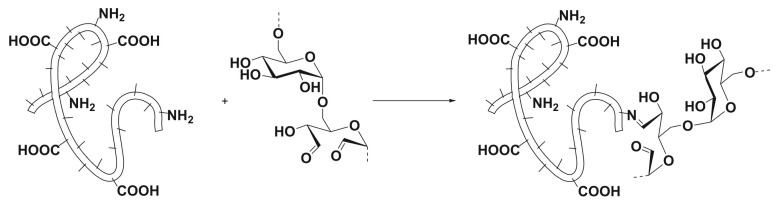
Schematic illustration of the cross-linking reaction between Dex-Ox and Gel.

Oxidized dextran (Dex-Ox) was used as a cross-linking agent so that high-energy light irradiation, toxic chemicals [46,47,48], or free radicals formation [29] was avoided. Initially, Dex was oxidized by sodium periodate [40,49], forming Dex-Ox with approximately 50 aldehyde groups per 100 units of the biopolymer chain. Comparing the ${}^{1}$H NMR spectrum of unmodified Dex to the spectrum of Dex-Ox (see Figure S1 in Supplement), in ${}^{1}$H NMR of Dex-Ox, several characteristic peaks were observed in the region of 6.0–4.4 ppm. These signals, which were assigned to protons of hemiacetals formed from aldehyde groups, confirmed the successful oxidation of Dex [40]. Subsequently, the hydrogels were obtained when Gels of bovine, rabbit, or chicken origin were chemically cross-linked by Dex-Ox, expecting Schiff base formation between Dex-Ox and amino groups present in Gel [50], as presented in Figure 1. The chosen manner of hydrogel preparation is characterized by mild conditions, avoiding the presence of harmful chemicals or radiation, which could be favorable for the intended application with respect to cell compatibility and viability.

### 3.2. Reaction Kinetics

Rheological experiments were designed to determine the kinetics of cross-linking reaction of Gels with Dex-Ox and a time of sol–gel transition, i.e., gelation point. A basic kinetics model of the first order was employed to fit the experimental rheological data and determine the reaction rates in order to facilitate reasonable kinetic data comparison (details of the data processing procedure are given in Supplementary Information). Table 1 summarizes the evaluated reaction rate coefficients together with the corresponding coefficients of determination. As can be seen, the reaction rates of a cross-linking reaction of Gel-B and Gel-R with Dex-Ox are similar; all were found to be in the range of 7–12 h${}^{-1}$. In contrast, the cross-linking reaction of Gel-C was significantly slower with reaction rates in the range of 1.0–2.5 h${}^{-1}$. The differences in reaction rates between Gel-B and Gel-R versus Gel-C can be most probably attributed to the different manufacturing procedures of the gelatines and resulting different properties of the used protein material. The rate coefficients show that conversion of cross-linking reaction equal to 75% is achieved in a time shorter than 10 min in case of Gel-B and Gel-R, while it took more than 30 min to reach this conversion in case of Gel-C. From a practical point of view, it is advantageous to print the hydrogel after reaching such conversion of cross-linking reaction in order to ensure the relatively stable properties of ink during printing.

We should note that in some cases, a short initial “lag” period was observed. This lag period can be attributed to the cross-linking reaction complexity. Despite this not being described by a first-order kinetic model, the overall fit was good, as is documented by the values of coefficients of determination and similar values of reaction rate coefficients of each type of Gel. As a result, even a simple model of the first order was able to acceptably describe the course of this reaction.

Another significant characteristic obtained in this measurement is the gelation point, which describes the solidification of the material and therefore can be found as the time when the crossing of storage and loss moduli occurs [51]. From that point, elastic forces begin to overcome the viscous ones, and the substance is defined as solid. The gelation point was not recorded in case of 1:1 polymer solution ratio for neither Gel-B nor Gel-R-based hydrogel (see Table 1) as the storage modulus is higher than the loss modulus; therefore, it is safe to assume that it is lower than 1 min, and gelation took place during the sample preparation. In case of Gel-C, the gelation point was detected after 2 min of reaction. When the proportion of Gel was increased, the gelation point increased as well to approximately 2 min. Curiously, no difference was detected between the Gel-B:Dex 2:1 and 3:1 solution ratios. However, Gel-R exhibits an additional 30 s increase in gelation time with each decrease of Dex-Ox content. Gel-C based gels exhibit the highest increase in gelation time. Despite this, all of the hydrogels solidify within 1 hour, which is rapid enough for their utilization in practice.

### 3.3. Rheology

Knowledge of hydrogels’ rheological behavior is of great importance in terms of printability and shape fidelity [52]. Cell viability can be ensured by minimizing the shear stress arising from the process [12]. A typical means of achieving this goal is to utilize a wider flow geometry [11]. However, this approach directly opposes precise positioning of the materials, which is the great advantage of 3D printing. Another way to reduce the shear stress during an ink flow is to reduce the viscosity [52]. However, during printing, a material with high viscosity and a significant difference between the loss and storage moduli is desirable due to the quickly achievable solid state. Other characteristics, such as brittleness of the extruded strand, also play an important role in the final appearance of the printed structure [42,52]. The above-mentioned requirements regarding the rheological behavior of gels indicate that the objectives of this work are to prepare a highly shear-thinning material with a fast sol–gel transition at various angular frequencies. For this purpose, the rheological properties of fully cross-linked hydrogels were characterized in the region of increasing angular frequency, simulating 3D-printing conditions.

To this end, the linear viscoelastic region (LVE) on a fully formed gel-like structure was checked at 35 ${}^{\circ }$C. Thus, strain sweep oscillatory measurements were performed with a constant angular frequency of 10 rad·s${}^{-1}$ for Gel:Dex-Ox 1:1 and 3:1 hydrogels (see Supplementary Information Figure S2). The LVE region was identified in the range where modulus G${}^{\prime }$ or G${}^{\prime \prime }$ is independent of the applied deformation from 0.1% to 100%. Thus, 10% deformation was used for the following frequency sweep oscillatory measurements. It is clear that besides Gel-R:Dex-Ox 3:1 and Gel-B:Dex-Ox 3:1, the storage modulus, G${}^{\prime }$, is the significant one describing a gel-like state.

**Figure 2:**
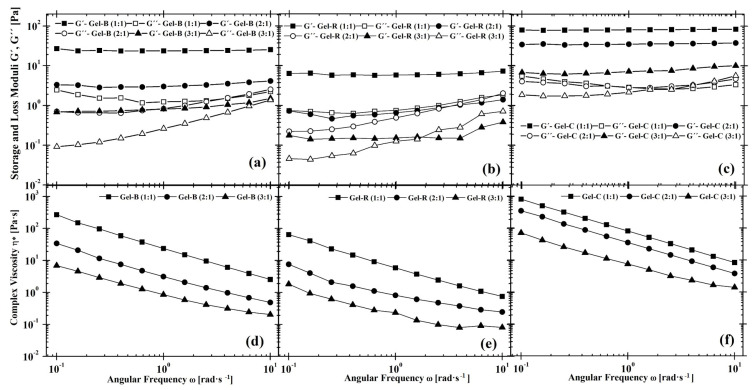
The angular frequency-dependent viscoelastic moduli (**a–c**) and complex viscosity (**d–f**) for Gel-based hydrogels: (**a**,**d**) Gel-B, (**b**,**e**) Gel-R, and (**c**,**f**) Gel-C for all examined Gel:Dex-Ox ratios.

This fact is also followed in the case of performed frequency sweep oscillatory measurements describing loss and storage modulus dependence on printing speed expressed by angular frequency. As can be seen in Figure 2, a strong shear-thinning behavior of the prepared hydrogels is observed. In case of all Gel-C:Dex-Ox solutions, the highest viscosities are reached as well as gel-like structure. It is clear that Gel-R:Dex-Ox solutions reach the lowest viscosity values. Moreover, in case of Gel-R:Dex-Ox 2:1; 3:1, and Gel-B:Dex-Ox 3:1 when the gelation point is taken into account, the sol-like structure is observed when complex viscosity is lower than 0.3 Pa·s. This means that the angular frequency at which the gelation point occurs depends on both Gel origin and biopolymer ratio. It should be mentioned that reverse measurements (from 10 to 0.1 rad·s${}^{-1}$) performed on the same sample achieved identical results as in the original measurements. Thus, any changes occurring in the material are reversible, even though such behavior is atypical in chemical hydrogels. Nevertheless, Khorsidi et al. [53] have found a growing number of amine-aldehyde cross-links to correlate with the increased shear-thinning character of hydrogels. Another research found that similar material compositions to the ones examined in the current study can be printed by microextrusion; therefore, a certain level of shear-thinning behavior can be assumed [54].

This described rheological characterization proves that the prepared hydrogels are suitable materials for 3D printing by microextrusion due to their shear flow and stability after stress relaxation. Based on experiments performed from prepared hydrogels composed of different gels and biopolymer ratios, it should be noted that these materials can be used in a variety of applications for 3D printing with specific rheological properties.

### 3.4. 3D Printing

Printability of the materials was practically assessed in microextrusion printing experiments. Two contributions to printing precision were measured—die swell and shape fidelity. Die swell is a parameter that affects the printing resolution, pore size, and layer height [14,55]. Several studies, both theoretical and experimental, have confirmed the significance of the phenomenon on the process of 3D printing [33,56,57,58,59,60,61]. Nevertheless, it is often omitted in the research of biopolymer based hydrogels as materials for microextrusion.

As is apparent from Table 2, all hydrogels examined in the current study experience a non-negligible die swell, reaching up to 3.3 times increase of the strand diameter. That is a clear indication of significant normal stress being built up in the material during shearing [14]. The die swell is notably lower in case of Gel-C:Dex-Ox 1:1 hydrogel. Some differences are found in the relative standard deviation (RSD) of die swell corresponding to different origins of Gel. The higher RSD suggests fluctuations in strand diameter, which are especially prominent in Gel-B-based materials and would consequently lead to lower printing precision. It can also indicate the phenomenon of over-gelation being present [42]. Based on this information, the highest printing precision is expected from Gel-C-based hydrogel. No significant difference caused by variation in Dex-Ox content was found, regardless of the Gel origin. It is possible that large fluctuations masked the influence of cross-linking agent amount on die swell.

The shape fidelity was characterized using the so-called printability (Pr) parameter, which was evaluated following a simple procedure described in [42]. This parameter reflects the precision of printing square-shaped pores. It is closely connected to the phenomenon of under- and over-gelation, and to a certain extend, it is able to describe the smoothness of the strand as well as hydrogel stability after removal of shear stress. The Pr values of most hydrogels are close to 1, as can be seen in Table 2, which encourages the possibility of using these materials for precise printing. Only Gel-B:Dex-Ox 3:1 exhibited insufficient mechanical strength of the strand, which caused the material to be completely fused and prevented the measurement. No significant difference in Pr with respect to neither Dex-Ox content nor Gel origin was observed.

Moreover, the printing of 5 layers of material proved that hydrogels presented in the current study provide self-supporting structures, i.e., those that do not collapse due to their own weight, in the 3D printing process. These structures were further used in the study of printing-induced porosity and hydrogel inner porosity.

**Figure 3:**
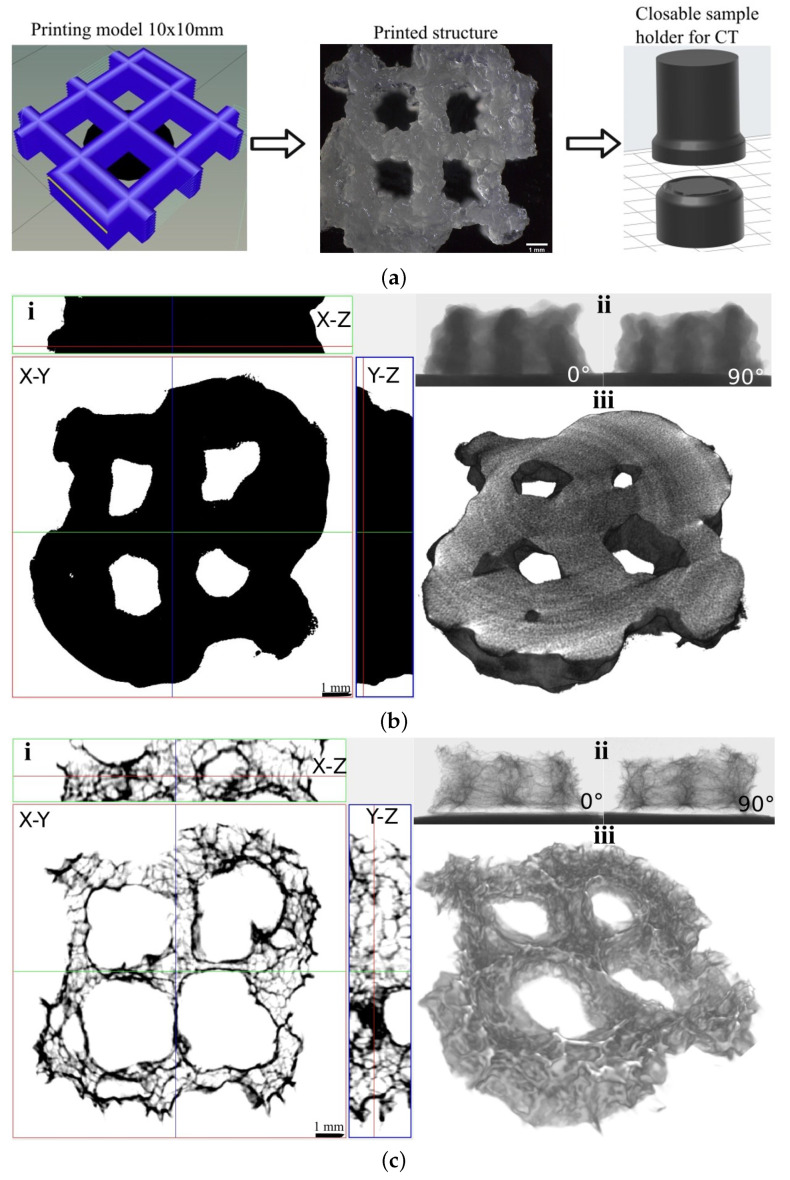
CT analysis of printed structures; (**a**) Scheme of sample preparation, (**b**) As-printed structure, (**c**) Lyophilized structure: **i**—2D cross-sections in respective planes, **ii**—X-ray adsorption for either 0${}^{\circ }$ or 90${}^{\circ }$, and **iii**—3D model.

To investigate the shape and pore distribution in the printed scaffold, the selected sample (Gel-C:Dex-Ox 1:1) was analyzed using CT (Figure 3). Due to the limited resolution of the CT used—SkyScan 1174 (6–30 μm per voxel), a special printing model was created for these purposes (Figure 3a left). In addition, it was necessary to create a special closed box for the hydrogel to prevent it from drying out during a 60-min CT scan (Figure 3a right). Figure 3b,c compares the scaffold in the hydrated state and after freeze drying. A comparison of these figures shows that the shape of the printed structure corresponds to the desired model both in the hydrated and dry state. The hydrated structure is larger than the freeze-dried structure and does not contain pores inside printed layers, detectable air bubbles, or other large defects. The printed hydrogel occupies 59% of the volume (299 mm${}^{3}$) with a surface area of 722 mm${}^{2}$. After lyophilization, the volume of the printed structure decreases to 63 mm${}^{3}$ (13% of space), while its surface increases to 1061 mm${}^{2}$ due to the formation of open pores. The analyzed space was 11.1 × 11.1 × 4.1 mm${}^{3}$ (505 mm${}^{3}$). From X-ray adsorption images for two different angles (0${}^{\circ }$ and 90${}^{\circ }$), it is clear that the printed material is accumulating in accordance with the printing model.

### 3.5. Swelling Tests

**Figure 4:**
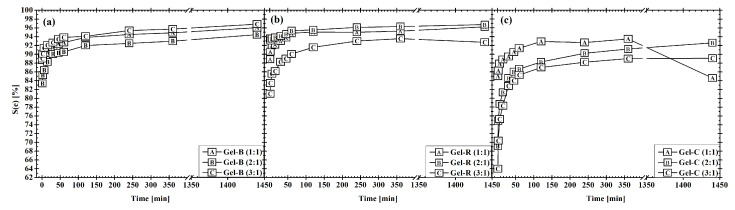
Swelling of Gel-based hydrogels: (**a**) Gel-B, (**b**) Gel-R, and (**c**) Gel-C.

Swelling is defined as the amount of buffer or water bound into a hydrogel. It is considered to be a crucial characteristic of hydrogels, as it gives an initial view of their hydrophilicity and cross-linking density. In general, rigid networks lead to lower water uptake [62]. Moreover, the swelling characterization is useful in the hydrogel preparation procedure as an insight into the possibility of cell proliferation or to determine hydrogel stability over time. Figure 4 shows the equilibrium swelling of prepared hydrogels at different ratios of Gel and Dex-Ox. The only observed difference between used Gels is the speed of PBS uptake, being notably lower in the case of Gel-C in comparison. Meanwhile, both Gel-B and Gel-R displayed similar swelling behavior. These results could refer to different network rigidity. This assumption was supported by rheology results (see Figure 2). Statistical analysis of the swelling test results proved that the concentration of the cross-linking agent has a minimal impact on swelling, and the observed differences correspond to the measurement deviation. Thus, the mechanical characteristics and rheology of the prepared hydrogel can be tailored without any impact on swelling, which is advantageous in the case of the cell proliferation.

### 3.6. Inner Porosity

SEM micrographs of the printed products after freeze drying revealed the highly porous inner structure of the materials. Table 3 presents the results of the average pore diameter in the cross-section. In addition, the relative pore area was determined in the cross-section in order to assess the porosity of the hydrogel. The pores are interconnected (see Supplementary Information Figure S2) and fall approximately in the range 50–100 μm in diameter [63]. The pore size remains practically constant regardless of both the Gel origin and the amount of Dex-Ox.

### 3.7. Cytotoxicity

**Figure 5:**
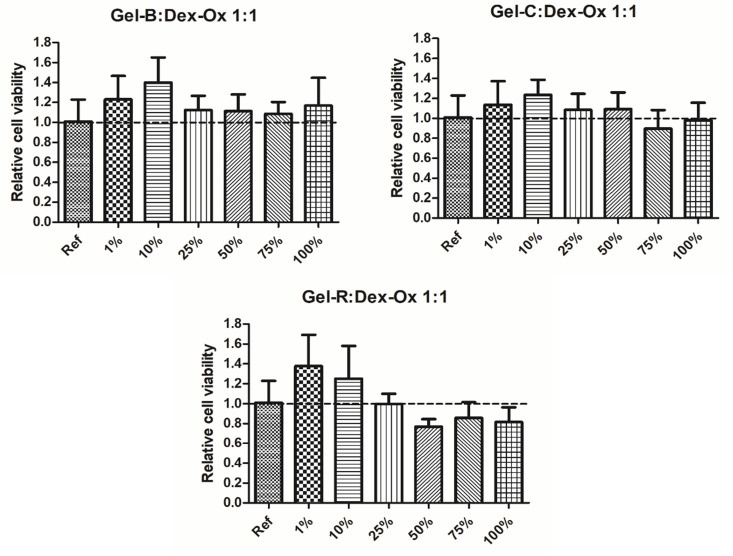
Cell viability determined by ATP assay performed on extracts from Gel:Dex-Ox 1:1 hydrogels.

Due to their natural origin, the chosen biopolymers—Gel and Dex—are generally characterized by low toxicity [26,64,65,66], which makes them especially advantageous for scaffold preparation. However, the presence of highly reactive aldehyde groups raises the concerns over the biocompatibility of Dex-Ox [66,67]. Additionally, the cytotoxicity of Dex-Ox has been observed to increase with the decrease of M${}_{w}$ [68]. In order to address this issue, the cytotoxicity of the here-prepared hydrogels was tested. Samples with the highest amount of potentially cytotoxic component, i.e., those with a solution ratio of 1:1, were chosen for the test. The results are presented in Figure 5. As can be seen, Gel-B and Gel-C were non-cytotoxic in a whole range of concentrations. A non-significant decrease in cell viability in observed in the case of Gel-R for a concentration of extract above 50%. However, the viability does not decrease below 70%, which is the limit of cytotoxicity potential. It can be concluded that all tested hydrogels do not express cytotoxicity potential. These results are highly encouraging in terms of using the proposed hydrogels, especially Gel-B and Gel-C-based ones, as bioinks for the preparation of scaffolds.

### 3.8. Cell Distribution within 3D-Printed Structure Evaluation

**Figure 6:**
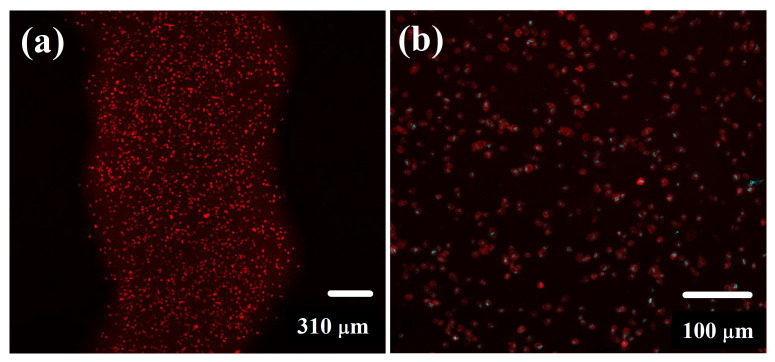
Microextruded Gel-B:Dex-Ox strand with incorporated mouse fibroblasts observed by the means of fluorescence confocal microscopy—(**a**) 4× magnification—image of cytoskeleton and (**b**) 10× magnification—overlay of cell nuclei and cytoskeleton images.

The homogeneity of cell distribution within the structure of scaffolds is a critical parameter for their applicability. This parameter is not ideal in case of the standard procedure of cell seeding into the scaffolds (e.g., by forcing the cells through a scaffold by either internal pressure or external vacuum pressure). The direct printing of cells within the material allows overcoming this problem. Thus, the effect of microextrusion on mouse fibroblasts distribution was observed by the means of LSCM. As can be seen in Figure 6, 3D printing ensured a homogeneous distribution of cells within the printed material. In addition, the overlay (Figure 6) demonstrated that cell nuclei were located inside undisturbed cells; thus, fibroblasts were not destroyed during 3D printing. The results are promising in terms of considering the presented biopolymers-based hydrogels as bioinks.

## 4. Conclusions

A series of hydrogels, which may potentially serve as bioinks, formed from Gel of different origin (bovine, rabbit, and chicken) cross-linked with various ratios of Dex-Ox were prepared by means of a simple and rapid method. Even though there are differences in Gel behavior depending on its origin, 3D-printing studies usually focus on bovine or porcine Gel, while research of rabbit and chicken Gel is rather scarce in this field. Study of the rheological behavior of the materials upon application of shear stress proved that the all investigated hydrogels were able to flow in shear, while they remain stable after stress relaxation and consequently are well suited for utilization in microextrusion. Additionally, die swell was significant, reaching a threefold increase in strand diameter in case of Gel-R and Gel-B samples. From the printing precision point of view, Gel-C was the most promising with the lowest die swell. Measurements confirmed that the complex viscosity of the hydrogels increased with the higher amount of cross-linking agent—Dex-Ox. In addition, rheology facilitated the study of reaction kinetics. This confirmed that the cross-linking reaction followed kinetics of the first order, and the gelation point was reached later as the amount of Dex-Ox solution decreased.

All the investigated hydrogels were able to form self-supporting structures in several layers, despite their various rheological properties. Moreover, the CT analysis confirmed that it is possible to produce constructs with continuous macroscopic pores throughout the structure via microextrusion processing of the hydrogels. In addition, the constructs remained stable even after freeze drying, and their highly porous inner structure was proved by means of CT and SEM measurements.

Optical imaging revealed that fluorescent-labeled mouse fibroblasts encapsulated within the polymeric matrix were of uniform distribution throughout the printed materials, and no cell disruption was observed. Finally, the printed constructs displayed no cytotoxicity in case of all tested materials. Thus, 3D-printable hydrogels with a potential to serve as bioinks have been successfully developed in the current study.

## Figures and Tables

**Table 1:** Dependence of reaction rate coefficient on reaction mixture composition

Gel:Dex-Ox Solution Ratio	Reaction Rate Coefficient (h${}^{-1}$)	Coefficient of Determination (1)	Gelation Point (min)
Gel-B			
1:1	11.6	0.998	<1
2:1	8.3	0.990	2
3:1	10.6	0.986	2
Gel-R			
1:1	11.1	0.993	<1
2:1	7.6	0.994	2
3:1	8.7	0.990	2.5
Gel-C			
1:1	2.0	0.997	2
2:1	1.2	0.999	13
3:1	2.4	0.994	>30

**Table 2:** Printing characteristics of Gel-based hydrogels.

Gel:Dex-Ox Solution Ratio	Die Swell (1)	Relative Standard Deviation (RSD) of Die Swell (%)	Printability (Pr) (1)
Gel-B			
1:1	3.0	13	1.0 ± 0.2
2:1	3.2	11	1.0 ± 0.2
3:1	3.0	14	/
Gel-R			
1:1	2.9	10	0.90 ± 0.09
2:1	3.3	6	0.873 ± 0.009
3:1	3.2	10	0.90 ± 0.07
Gel-C			
1:1	2.4	7	1.0 ± 0.1
2:1	2.6	9	1.0 ± 0.2
3:1	2.7	8	0.92 ± 0.09

**Table 3:** Evaluation of pore size and porosity of hydrogels after shear strain and subsequent lyophylization.

Gel:Dex-Ox Solution Ratio	Average Pore Size (mm${}^{2}$)	Relative Pore Area (%)
Gel-B		
1:1	0.014 ± 0.009	40–70
2:1	0.017 ± 0.005	45–80
3:1	0.020 ± 0.009	60–75
Gel-R		
1:1	0.017 ± 0.006	40–80
2:1	0.011 ± 0.003	35–65
3:1	0.009 ± 0.004	35–50
Gel-C		
1:1	0.010 ± 0.004	35–45
2:1	0.036 ± 0.008	45–50
3:1	0.04 ± 0.02	30–50

## Data Availability

The data presented in this study are available on request from the corresponding author.

## Supplementary Materials

The following are available at https://www.mdpi.com/article/10.3390/polym14030391/s1, Figure S1: 1H NMR spektra of dextran (Dex) and dextran after oxidation (Dex-Ox), Figure S2: Linearity sweep for hydrogels with different amount of cross-linking agent, Figure S3: SEM micrograph of lyophilized hydrogels in cross section
