# Supplementary material for: Cross-Linked Gelatine by Modified Dextran as a Potential Bioink Prepared by a Simple and Non-Toxic Process
Source: Polymers (Basel). 2022 Jan 19;14(3):391. doi: 10.3390/polym14030391 (PMC8838658; doi:10.3390/polym14030391)
Supplement: Supplementary file 1 [file polymers-14-00391-s001.zip › polymers-1540309-supplementary.pdf]

# Cross-linked Gelatine by Modified Dextran as a Novel Bioink Prepared by a Simple and Non-toxic Process - Supporting Information

**Dr. L. Musilová, Dr. E. Achbergerová, L. Vítková, Dr. R. Kolařík, M. Kašparová, Prof. A. Minařík, Prof. A. Mráček, Prof. P. Humpolíček, Dr. J. Pecha**

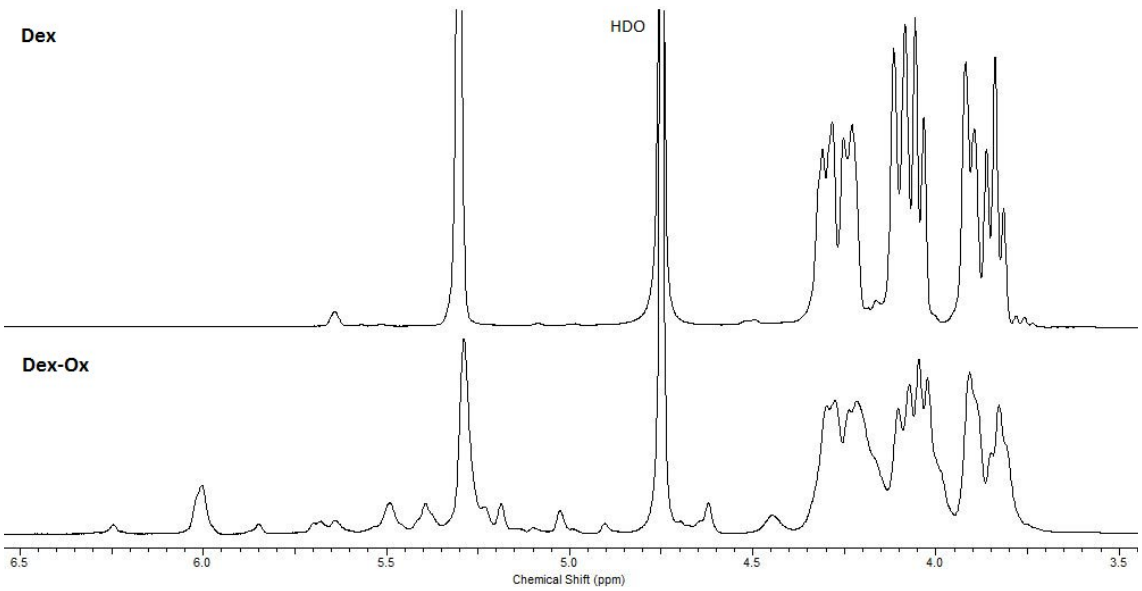


Figure S1: 1H NMR spektra of dextran (Dex) and dextran after oxidation (Dex-Ox)

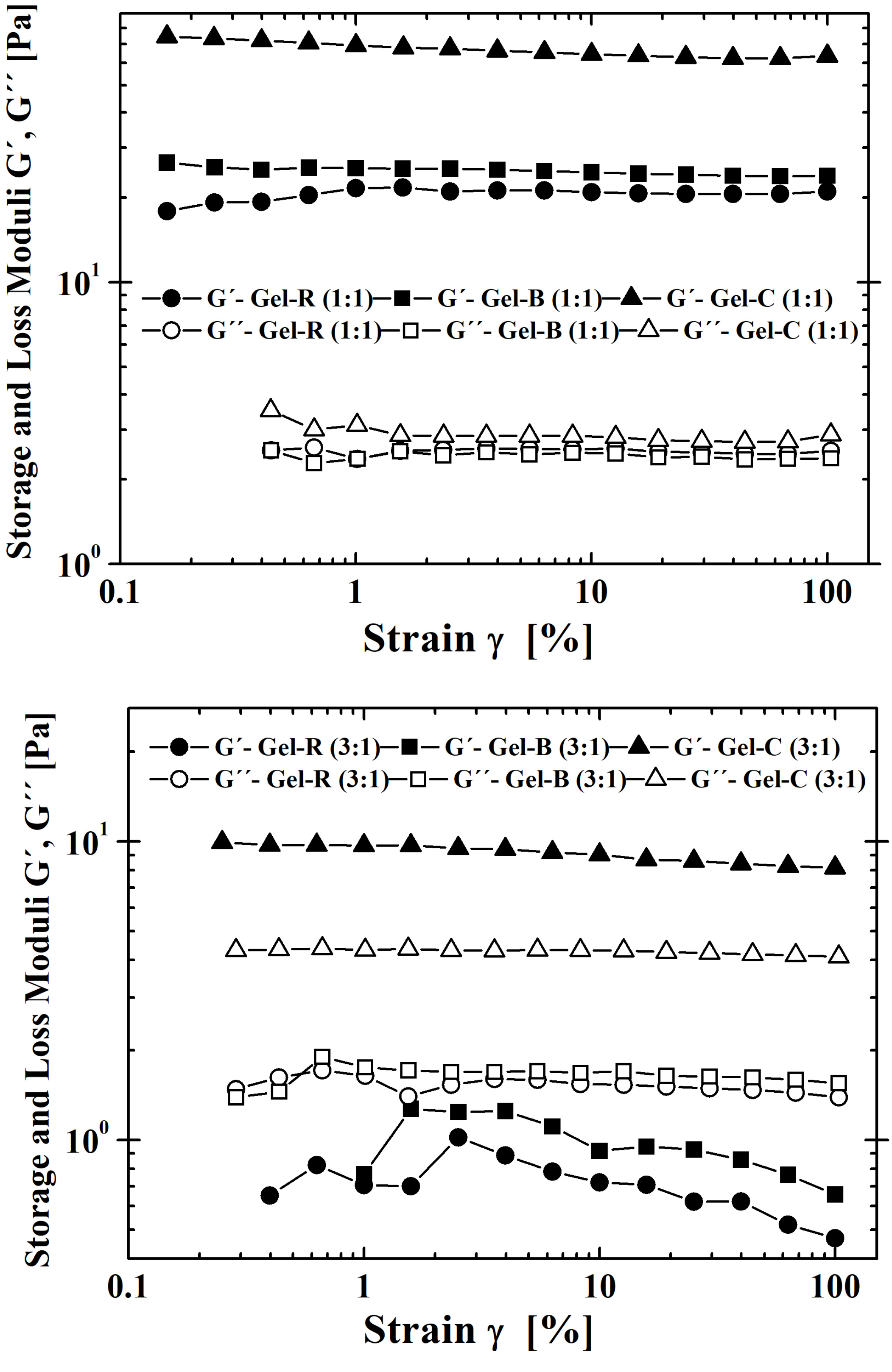


Figure S2: Linearity sweep for hydrogels with different amount of cross-linking agent

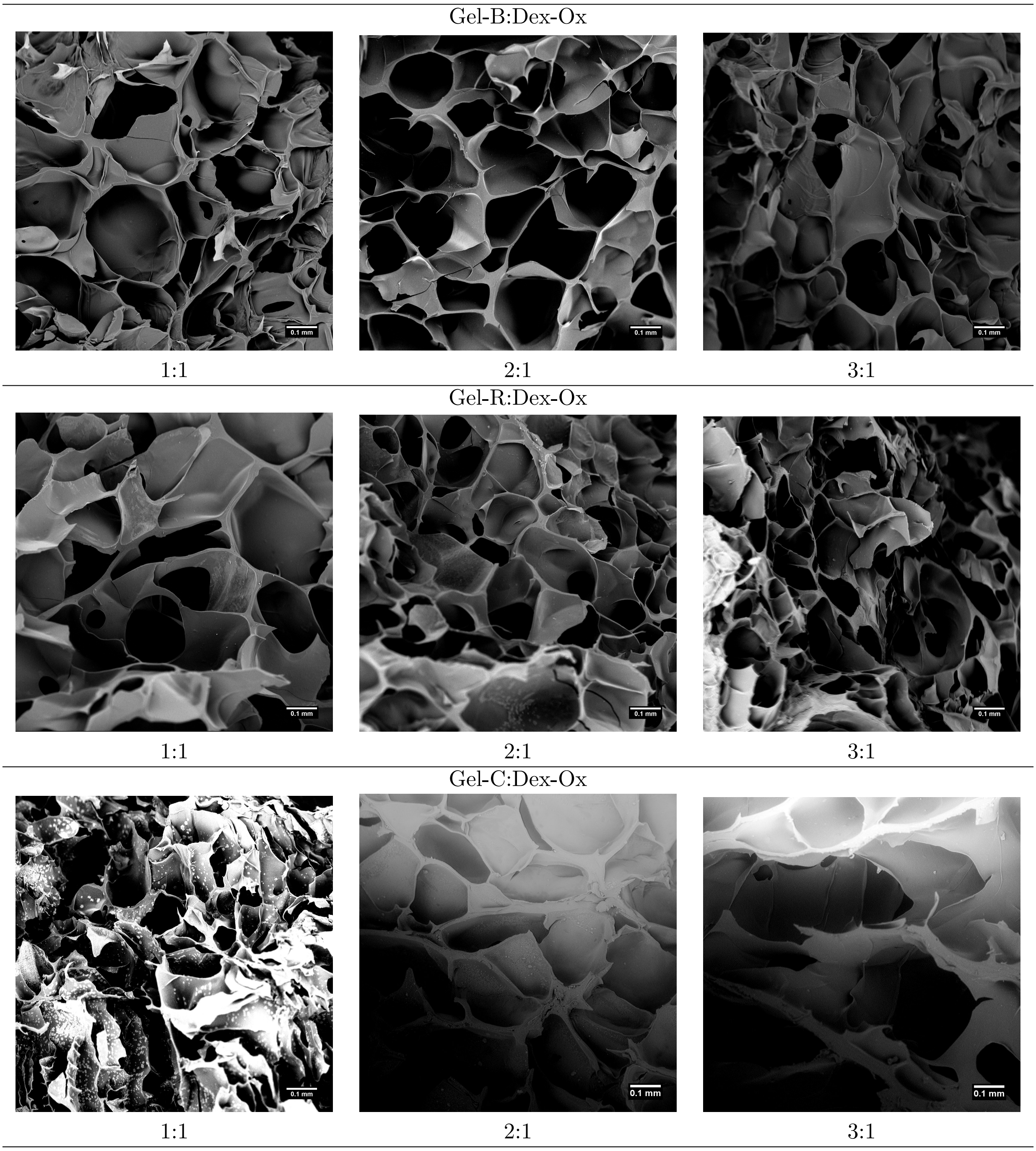


Figure S3: SEM micrograph of lyophilized hydrogels in cross section

## Kinetics of Cross-linking Reaction - Methodology of Evaluation

A cross-linking reaction between aldehyde groups of oxidized dextran and reactive groups of Gel (preferably free amino groups [Hoffman et al., 2004]) takes place during hydrogel formation. The reaction overall stoichiometry can be written simply as:

$$A \xrightarrow{\text{cross-linking}} B \tag{1}$$

where A stands for concentration of unreacted groups involved in the reaction (either at the side of oxidized dextran or at the side of gelatine) and B for the newly formed bonds (i.e. cross-linking reaction products). The cross-linking reaction of proteins itself is complex and complicated, involves several reaction steps including parallel and consecutive reactions and is not completely understood even in case of relatively simple cross-linking agents like glutaraldehyde [Migneault et al., 2018; Stefano et al., 2010]. Consequently, we applied relatively simple approach for evaluation of cross-linking kinetics, the purpose of which was to follow evolution of the overall reaction and assess possible differences in the cross-linking reaction between investigated samples of protein materials (gelatines) and oxidized dextran.

Complex viscosity was used as a proxy for concentration of reacted groups (B) and their evolution in time. Its initial value ($\eta_0^*$) characterizes initial concentration of reacted groups ($c_{B0}$) given by the delay in viscosity measurements after mixing of both reactants and includes also viscosity of all reactants themselves. Estimation of equilibrium (i.e. final) viscosity ($\eta_\infty^*$) served for estimation of maximal achievable concentration of reacted groups ($c_{B\infty}$) at the applied reaction conditions which is equivalent to the overall initial concentration of accessible reaction groups of the reactant ($c_{A0}$).

We employed basic kinetics model of the first order to fit the experimental data. Taking into account presented reaction stoichiometry and assumed simplifications, the model of the first order can be written in terms of complex viscosity as follows:

$$\frac{d\eta^*}{d\tau} = k \cdot (\eta_\infty^* - \eta^* \tau) \tag{2}$$

where k stands for reaction rate coefficient and $\tau$ for time.

Reaction rate coefficients k were evaluated from the linearized form of the integrated model (2) in a standard manner.

# References


1. A. S. Hoffman and F. J. Schoen and J. E. Lemons.*Biomaterials Science: An Introduction to Materials in Medicine*. Elsevier Academic Press, London. **2004**. ISBN: 0-12-582463-7
2. I. Migneault and C. Dartiguenave and M. J. Bertrand and K. C. Waldron. *Glutaraldehyde: behavior in aqueous solution, reaction with proteins, and application to enzyme crosslinking*.Biotechniques. **2018**. DOI: 10.2144/04375RV01
3. F. Stefano and J. Song and Q. Huang.*Alternative reaction mechanism for the cross-linking of gelatin with glutaraldehyde*. Journal of agricultural and food chemistry. **2010**. DOI: 10.1021/jf9031603
